# Long‐term quality of life after definitive treatment for prostate cancer: patient‐reported outcomes in the second posttreatment decade

**DOI:** 10.1002/cam4.1103

**Published:** 2017-05-31

**Authors:** Joanne W. Jang, Michael R. Drumm, Jason A. Efstathiou, Jonathan J. Paly, Andrzej Niemierko, Marek Ancukiewicz, James A. Talcott, Jack A. Clark, Anthony L. Zietman

**Affiliations:** ^1^ Beth Israel Deaconess Medical Center Boston Massachusetts; ^2^ Department of Radiation Oncology Massachusetts General Hospital Harvard Medical School Boston Massachusetts; ^3^ Division of Biostatistics Department of Radiation Oncology Massachusetts General Hospital Boston Massachusetts; ^4^ Department of Medical Oncology Continuum Cancer Centers of New York New York New York; ^5^ Center for Health Quality Outcomes and Economic Research Edith Nourse Rogers Memorial Veterans Hospital Bedford Massachusetts

**Keywords:** Long‐term quality of life, Patient‐reported outcomes, prostate cancer, treatment choice, treatment‐related dysfunction

## Abstract

Definitive treatment for prostate cancer includes radical prostatectomy (RP), external beam radiation therapy (EBRT), and brachytherapy (BT). The different side effect profiles of these options are crucial factors for patients and clinicians when deciding between treatments. This study reports long‐term health‐related quality of life (HRQOL) for patients in their second decade after treatment for prostate cancer. We used a validated survey to assess urinary, bowel, and sexual function and HRQOL in a prospective cohort of patients diagnosed with localized prostate cancer 14–18 years previously. We report and compare the outcomes of patients who were initially treated with RP, EBRT, or BT. Of 230 eligible patients, the response rate was 92% (*n* = 211) and median follow‐up was 14.6 years. Compared to baseline, RP patients had significantly worse urinary incontinence and sexual function, EBRT patients had worse scores in all domains, and BT patients had worse urinary incontinence, urinary irritation/obstruction, and sexual function. When comparing treatment groups, RP patients underwent larger declines in urinary continence than did BT patients, and EBRT and BT patients experienced larger changes in urinary irritation/obstruction. Baseline functional status was significantly associated with long‐term function for urinary obstruction and bowel function domains. This is one of the few prospective reports on quality of life for prostate cancer patients beyond 10 years, and adds information about the late consequences of treatment choices. These data may help patients make informed decisions regarding treatment choice based on symptoms they may experience in the decades ahead.

## Introduction

Definitive treatment options for localized prostate cancer include radical prostatectomy (RP), external beam radiation therapy (EBRT), and brachytherapy (BT). There are no randomized trials comparing all three treatment modalities, and no treatment option has been shown to be more effective at cancer control than the others 1, 2. Regardless of treatment choice, patients with localized prostate cancer generally have a favorable prognosis that appears to be predicted more by pretreatment pathologic and disease factors than by treatment 3.

Most men diagnosed with prostate cancer die of causes other than this disease 4. As a result, patients treated for prostate cancer can expect to live with the long‐term side effects of their treatment for many years, even decades, before dying of other causes. Consequently, the side effect profiles of the different treatments should play a central role in the decision‐making process for patients and their physicians.

Many studies have clearly documented early posttreatment HRQOL outcomes: urinary incontinence and sexual dysfunction after RP, urinary irritative and obstructive symptoms, bowel problems, delayed sexual dysfunction after EBRT or BT, and long‐term urinary incontinence after BT. There are, however, few studies that examine or compare HRQOL after different treatments for prostate cancer for more than the first few years, which is too short to document the known late effects of radiation 5, 6, 7, 8, 9, 10, 11. The consequences of a prior prostatectomy as men enter more advanced age is unknown. Reported studies, therefore, provide an incomplete picture of what patients should expect following treatment. The goal of this study was to examine the long‐term changes in urinary incontinence, urinary irritation/obstruction, bowel, and sexual domains at least 10 years after definitive treatment for patients with prostate cancer.

## Methods

### Patient population

Patients with untreated localized prostate cancer were recruited between 1994 and 2000 when they were seen for consultation at Massachusetts General Hospital, Dana‐Farber Cancer Institute/Brigham and Women's Hospital, Beth‐Israel Deaconess Medical Center, or Metro West Medical Center. We did not require that their treatment occur at one of the recruitment sites, but enrolled patients participated in a prospective observational study of patient‐reported outcomes and quality of life after definitive treatment for prostate cancer, which has been previously described 5, 10, 11, 12. Briefly, the study monitored urinary, bowel, and sexual quality of life at baseline, and at 3, 12, 24, and 36 months after initiating treatment.

In our report in 2006, 338 enrolled patients were still alive with current contact information 12. As of December 2011, 100 of these patients were known to have died and eight could no longer be found. As a result, new follow‐up questionnaires were mailed to a total of 230 patients, along with a $10 CVS card. Patients who did not return questionnaires within 3 weeks were called on the phone as a reminder, and given a chance to refuse participation. Our institutional review board approved this study. The investigators obtained informed consent from each participant.

### HRQOL instruments and data collection

We used a validated instrument, the Prostate Cancer Symptom Indices (PCSI), to measure function in the domains of urinary incontinence, urinary irritation/obstruction, bowel function, and sexual function as previously described 13. Each functional index was scored from 0 to 100, with higher scores indicating worse function. In order to compare baseline functional status to current functional status, we correlated these function indices with patient‐reported levels of distress to categorize patients into either “normal” or “abnormal” functional groups as previously described 5, 14. The follow‐up questionnaire also included the symptom bother scales of the Expanded Prostate Index Composite 15, and the Physical Component Summary (PCS) and Mental Component Summary (MCS) of the SF‐12 16.

Pretreatment prostate‐specific antigen (PSA), Gleason score (GS), cancer stage 17, modality of primary treatment, and patient demographic factors had all been collected previously by chart review. Medical comorbidities were scored using the Index of Co‐Existent Disease (ICED) 18.

### Statistical considerations

Patient demographic and clinical characteristics were compared using Pearson's chi‐squared test for categorical variables and Kruskal–Wallis test for continuous variables. Change scores were defined as the difference between baseline functional score and current functional score. Change scores were compared between different treatment groups using the Wilcoxon rank‐sum test.

For each domain, we used multiple linear regression analysis to determine demographic and clinical factors associated with worse change scores, including treatment modality, age, race, ICED score, baseline and current PCS, and baseline and current MCS. We applied rank ‐transformation to change scores, as the distribution of change scores was strongly non‐normal. We excluded records with missing data and used Wald tests for assessing statistical significance. As baseline functional group was highly correlated with change score, it could not be used in the same model to assess its impact.

To assess the impact of baseline functional group, we used a linear model to analyze the effect of treatment modality and baseline functional group on the current functional score in each domain. In order to directly compare all three treatment modalities, we performed a pairwise comparison of marginal linear predictions. All analyses were performed without adjustment for multiple comparisons.

Analyses were performed using Stata software (version 14.1; College Station, TX). Results were considered statistically significant when the *P*‐value was <0.05, and all reported *P*‐values are two‐sided.

## Results

**Figure 1:**
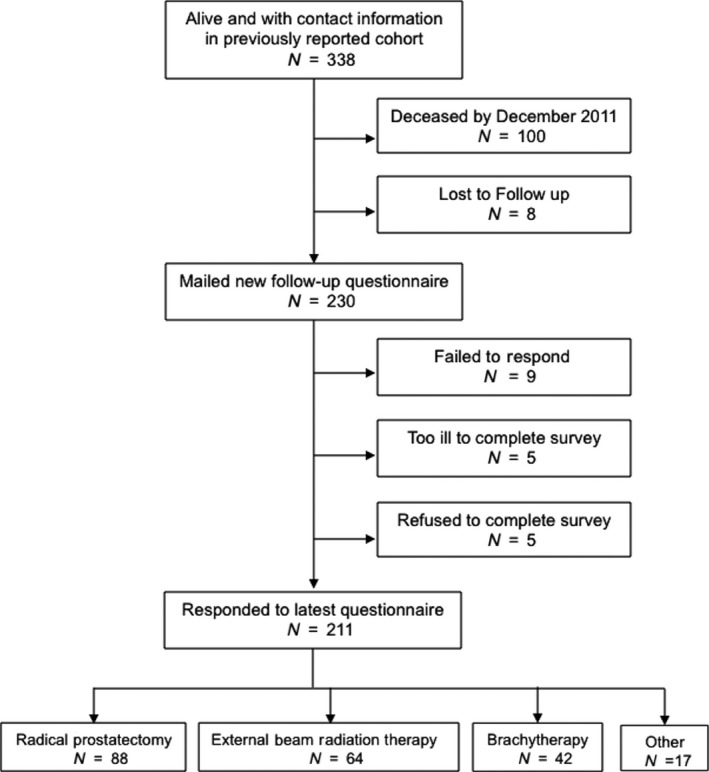
Patient flow chart that describes patients in the cohort, subsequent attrition, and treatment choice.

Of 230 patients who were mailed questionnaires, five refused, five were too ill to complete the survey, and nine failed to respond. Thus, a total of 211 patients (92%) responded to the latest follow‐up questionnaire (Fig. 1). We compared the baseline age, education, marital status, income level, employment status, baseline PCS, baseline MCS, ICED score, baseline function groups in the urinary incontinence, urinary irritation/obstruction, bowel, and sexual domains, pretreatment PSA, Gleason score, clinical stage, and primary treatment between the responders and nonresponders. Nonresponders were slightly older (*P* = 0.023), had lower income (*P* = 0.044), and were less likely to be employed (*P* = 0.047) prior to treatment compared to responders.

Baseline demographic and clinical characteristics for all responding patients separated by primary treatment are shown in Table 1. Forty‐two percent of patients had had RP, 30% EBRT, and 20% low‐dose‐rate (LDR) BT. Nine percent of the patients receiving EBRT also had a BT boost. The remaining 8% of patients underwent observation or had other modalities of primary treatment, such as cryotherapy or androgen deprivation therapy (ADT). Due to the small number of patients receiving observation or other treatments, our analysis is limited to the 194 patients receiving RP, EBRT, or BT. In general, patients who underwent EBRT were older (*P* < 0.001), less likely to be employed (*P* = 0.030), had lower income (*P* = 0.010), higher pretreatment PSA (*P* = 0.003), and higher clinical stage (*P* < 0.001) at baseline, compared to patients who underwent RP or BT. Patients who underwent RP had lower MCS scores (0.041), higher Gleason score (*P* = 0.005), and longer follow‐up (*P* < 0.001) compared to patients who had EBRT or BT.

**Table 1:** Baseline demographic and clinical characteristics of responding patients

Characteristic	RP	EBRT	BT	*P*
*N*	88	64	42	
Age at treatment (years)				<0.001
Median (IQR)	59 (53–63)	67 (60–70)	63 (56–68)	
Age at last follow‐up (years)				<0.001
Median (IQR)	75 (69–79)	82 (75–85)	77 (69–81)	
Race (%)				0.14
White	99%	94%	88%	
Non‐white	1%	3%	5%	
Unknown	0%	3%	7%	
Education (%)				0.40
Completed college	89%	91%	79%	
Married (%)	92%	88%	83%	0.19
Employed (%)	66%	55%	76%	0.030
Annual household income (%)				0.010
≤$40,000	20%	27%	7%	
$40,000–$70,000	23%	36%	31%	
≥$70,000	52%	27%	48%	
Unknown	5%	11%	14%	
ICED (%)				0.32
1	35%	34%	26%	
2	56%	61%	62%	
3–4	0%	3%	0%	
Unknown	9%	2%	12%	
Physical component score				0.064
Median (IQR)	56 (52–58)	55 (50–57)	56 (53–57)	
Mental component score				0.041
Median (IQR)	52 (45–57)	55 (47–58)	56 (50–58)	
Pretreatment PSA				0.003
Median (IQR)	6 (5–9)	7 (5–12)	6 (5–7)	
Gleason score				0.005
Median (IQR)	7 (6–7)	6 (6–7)	6 (6–6)	
Clinical stage (%)				<0.001
T1, a–c	45%	34%	86%	
T2, a–c	36%	45%	2%	
T3, a–c	5%	20%	0%	
Unknown	14%	0%	12%	
Follow‐up since treatment (years)				<0.001
Median (range)	15.4 (13.5–17.6)	14.8 (12.6–17.6)	13.7 (12.7–16.4)	

Note: Although 211 patients responded to our survey, the analysis was limited to the 194 patients who had RP, EBRT, or BT. RP, radical prostatectomy; EBRT, external beam radiation therapy; BT, brachytherapy; IQR, interquartile range; ICED, index of co‐existent disease; PSA, prostate‐specific antigen.

At 12 to 18 years after treatment, patients’ median ages were 75, 82, and 77 for RP, EBRT, and BT, respectively. When patients were split by treatment group, patients who underwent RP had significantly worse current scores compared to baseline in the urinary incontinence (*P* < 0.001) and sexual function (*P* < 0.001) domains (Table 2). Patients that underwent EBRT had significantly worse current scores compared to baseline in the urinary incontinence (*P* < 0.001), urinary irritation/obstruction (*P* < 0.001), bowel function (*P* = 0.025), and sexual function (*P* < 0.001) domains. Patients who had BT had significantly worse current scores compared to baseline in the urinary incontinence (*P* = 0.007), urinary irritation/obstruction (*P* = 0.004), and sexual function (*P* < 0.001) domains.

**Table 2:** Comparison of current scores to baseline scores in all functional domains

Domain	RP (*N* = 88)	EBRT (*N* = 64)	BT (*N* = 42)	RP versus EBRT change score *P*‐value	RP versus BT change score *P*‐value	EBRT versus BT change score *P*‐value
Urinary incontinence						
Baseline median (IQR)	0 (0–0)	0 (0–0)	0 (0–0)			
Current median (IQR)	30 (0–42)	29 (0–39)	0 (0, 30)			
Change score median (IQR)	30 (0–42)	11 (0, 30)	0 (0, 30)	0.25	0.001	0.062
Baseline versus Current score *P*‐value	<0.001	<0.001	0.007			
Urinary Obstruction						
Baseline median (IQR)	18 (12–32)	17 (12–25)	17 (9–25)			
Current median (IQR)	20 (13–30)	23 (18–32)	25 (18–32)			
Change score median (IQR)	0 (−10–10)	6 (−2–13)	4 (−2–13)	0.005	0.010	0.83
Baseline versus current score *P*‐value	0.780	<0.001	0.004			
Bowel function						
Baseline median (IQR)	0 (0–8)	4 (0–8)	0 (0–8)			
Current median (IQR)	4 (0–8)	4 (0–12)	4 (0–8)			
Change score median (IQR)	0 (0–4)	0 (−4–8)	0 (0–4)	0.25	0.47	0.66
Baseline versus Current score *P*‐value	0.056	0.025	0.092			
Sexual function						
Baseline median (IQR)	30 (29–32)	30 (28–47)	30 (25–43			
Current median (IQR)	86 (44–100)	100 (77–100)	90 (40–100)			
Change score median (IQR)	28 (10–65)	58 (6–70)	31 (5–67)	0.43	0.81	0.48
Baseline versus Current score *P*‐value	<0.001	<0.001	<0.001			

Note: Although 211 patients responded to our survey, the analysis was limited to the 194 patients who had RP, EBRT, or BT. RP, radical prostatectomy; EBRT, external beam radiation therapy; BT, brachytherapy; IQR, interquartile range.

When comparing treatment modalities, change scores in the urinary incontinence domain were significantly worse for RP than for BT (*P* = 0.001), and change scores in the urinary irritation/obstruction domain were significantly worse for EBRT or BT than for RP (*P* = 0.005, *P* = 0.010, Table 2). There were no significant differences between treatment groups in the bowel function domain, and all treatment groups experienced similarly large differences in sexual function scores.

Clinically relevant predictor variables were entered into multivariate regression models to determine which factors were associated with change scores in the different domains. There were no variables significantly associated with a higher urinary incontinence change score (Table 3). Race (*P* = 0.011) was significantly associated with a higher urinary obstruction change score. For bowel function, a lower baseline MCS score (*P* = 0.009) and current MCS score (*P* = 0.037) were significantly associated with a higher change score. Finally, increased age at baseline (*P* = 0.038) was significantly associated with a higher sexual function change score. When directly comparing treatment modalities with a pairwise comparison of marginal linear predictions, and adjusting for covariates using the same multivariate model (Table 4), the average change score in urinary incontinence for RP was significantly worse than for BT (*P* = 0.005), and the average change scores in the urinary irritation/obstruction domain for EBRT or BT were significantly worse than for RP (*P* = 0.003, *P* = 0.013).

**Table 3:** Multivariate analysis of demographic and clinical variables on change scores

Covariates	Urinary incontinence	Urinary obstruction	Bowel function	Sexual function
Age	0.687	0.075	0.602	0.038
Race	0.388	0.011	0.720	0.169
ICED score	0.817	0.849	0.969	0.228
Baseline PCS	0.076	0.114	0.672	0.079
Baseline MCS	0.321	0.473	0.009	0.391
Current PCS	0.255	0.274	0.193	0.367
Current MCS	0.057	0.249	0.037	0.225

ICED, index of co‐existent disease; PCS, physical component summary; MCS, mental component summary.

**Table 4:** Pairwise comparison of treatment modalities, adjusting for covariates

Domain	EBRT compared to RP	BT compared to RP	BT compared to EBRT
Urinary incontinence			
Contrast	−5.23	−16.00	−10.80
*P*‐value	0.307	0.005	0.077
Confidence interval	−15.3, 4.85	−27.1, −4.91	−22.7, 1.17
Urinary obstruction			
Contrast	8.58	8.04	−0.53
*P*‐value	0.003	0.013	0.876
Confidence interval	2.89, 14.3	1.74, 14.3	−7.26, 6.20
Bowel function			
Contrast	1.01	1.68	0.67
*P*‐value	0.464	0.276	0.686
Confidence interval	−1.70, 3.72	−1.35, 4.71	−2.60, 3.94
Sexual function			
Contrast	2.70	1.84	−0.86
*P*‐value	0.677	0.800	0.912
Confidence interval	−10.1, 15.4	−12.5, 16.1	−16.1, 14.4

RP, radical prostatectomy; EBRT, external beam radiation therapy; BT, brachytherapy.

**Figure 2:**
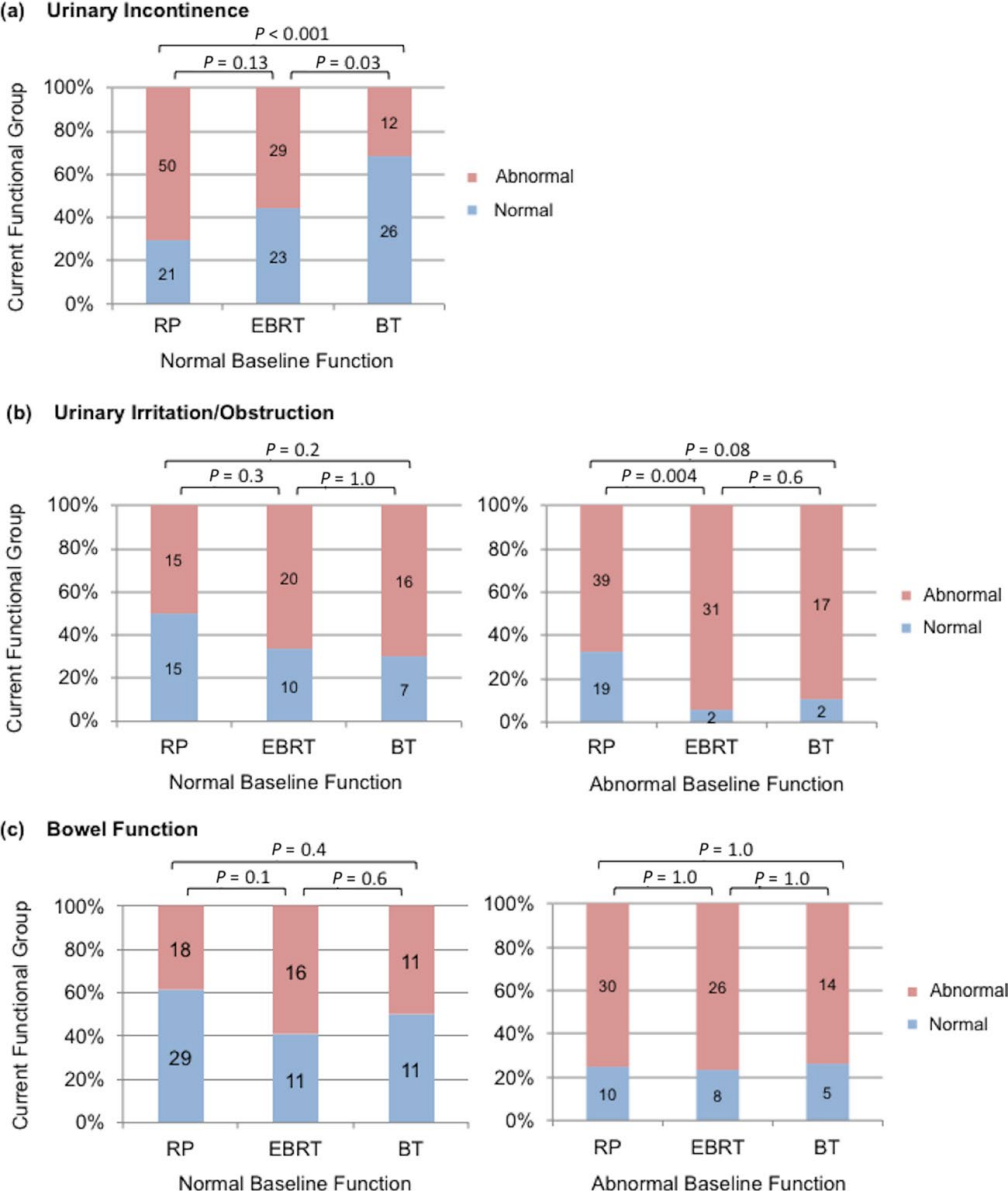
Current functional group stratified by baseline functional group, for urinary incontinence, urinary irritation/obstruction, and bowel function domains. Some data not shown because of insufficient data for those with abnormal baseline urinary incontinence and because of uniform abnormal current sexual functional status.

In a regression model including baseline functional group and current functional score, baseline functional group was directly correlated with current function score in the domains of urinary irritation/obstruction (*P* < 0.001, data not shown) and bowel function (*P* < 0.001), but not urinary incontinence (*P* = 0.174) or sexual function (*P* = 0.088). To explore this relationship further, Figure 2 displays the current functional group stratified by baseline functional group for each treatment modality. For urinary incontinence, abnormal functional outcomes seem more common in RP patients compared to BT patients with normal baseline function (*P* < 0.001), with insufficient data to examine patients with abnormal baseline function. In the urinary irritation/obstruction domain, RP patients were more likely to improve than EBRT patients if they started out with abnormal function (*P* = 0.004). Patients who received EBRT or BT were not more likely to have abnormal current bowel function as compared to those who received RP. Finally, all patients currently have abnormal sexual function regardless of treatment modality or baseline sexual functional group.

## Discussion

More than ever, treatment of localized prostate cancer is being reserved for patients with life expectancies of over 10 years. This is one of few studies that prospectively assessed urinary, bowel, and sexual quality of life in the second decade after primary treatment for prostate cancer, with a median follow‐up of nearly 15 years. As a result, this study contributes valuable information for patients and their caregivers when choosing between treatment options for prostate cancer.

We show that the three treatment options differ in long‐term side effect profiles. While there were detriments in urinary incontinence and sexual function for all patients regardless of treatment group, impairments in urinary irritation/obstruction compared to baseline were seen only for patients who underwent EBRT or BT, and a decrease in bowel function was seen only for patients who underwent EBRT. Moreover, long‐term change in urinary incontinence was worse for patients treated with RP compared to BT, while long‐term change in urinary irritation/obstruction was worse for patients treated with EBRT or BT when compared to RP.

The short‐ to intermediate‐term quality of life of patients treated for prostate cancer by different modalities has been well reported. Many studies have described differences in functional declines after RP, EBRT, and BT, including worse urinary incontinence following RP, worse urinary irritation/obstruction after BT, worse bowel symptoms following radiation therapy, and declines in sexual functioning after EBRT and RP 5, 6, 7, 8, 9, 19, 20, 21, 22, 23, 24, 25, 26, 27, 28. The ProtecT trial reported worse sexual function and urinary incontinence after RP and mostly transient worsening in sexual, bowel, and urinary irritative/obstructive symptoms after EBRT, which is consistent with previously published literature 29. Although this trial is groundbreaking in that it randomized a large number of patients to observation, RP, or EBRT, they have reported HRQOL data for only 6 years posttreatment, and did not include patients who received BT 29. Additionally, two large prospective studies have recently reported the short‐term HRQOL outcomes of patients treated with EBRT, RP, BT, or active surveillance. With 2 and 3 years of follow‐up, these studies found that patients experienced worse sexual function and urinary incontinence after RP, worse urinary irritative/obstructive symptoms after radiation therapy, and mostly transient declines in bowel function after EBRT 30, 31.

In addition, some studies have shown that baseline function is one of the most important predictors of final function in each domain 5, 10, 22, which we also found in our study for the urinary irritation/obstruction and bowel function domains. Surprisingly, with long‐term follow‐up, age was the only significant predictor of change in sexual function from baseline in our study, while baseline functional status was not. It is likely that with increasing time, the effects of age predominate over the effects of baseline status on sexual function after treatment for prostate cancer. That is, if their lifespan is sufficiently long, all men may develop severe sexual dysfunction regardless of treatment for prostate cancer. Some studies have documented sexual and urinary dysfunction in an aging population of men with no prostate cancer 32, 33, though any declines remain less severe than those experienced by men receiving treatment for prostate cancer 34. For urinary incontinence, only treatment with brachytherapy was a significant predictor of final function.

There have been few studies examining long‐term quality of life outcomes, and our results add novel observations. Additionally, many of these studies have limitations that this analysis addresses. Some have either no baseline measures or depended upon retrospective recall of baseline quality of life 20, 21, 27, had poor response rates after 10 years posttreatment 21, 26, utilized surveys that had little to no focus on urinary irritation/obstructive symptoms 20, 21, 26, 27, or did not include patients receiving brachytherapy 27.

Like a majority of these studies with longer follow‐up, we found that the disparity in urinary incontinence between RP patients and radiation patients persists into the second decade following treatment 20, 21, 26. Our finding that there is no long‐term difference in sexual function between patients receiving different treatments is also consistent with other long‐term reports 20, 26, 27, with all patients having poor sexual function and experiencing similarly large decreases from baseline. This may represent subsequent treatment, such as ADT for cancer progression, or may be a result of normal and inevitable functional loss with aging 34. For bowel function, only one study reported no long‐term difference in bowel function between radiation and RP 27. Our study also shows that by 15 years after treatment, there is no significant difference in the change in bowel dysfunction between treatment groups. This may be due to salvage EBRT, baseline bowel dysfunction of elderly men, or a combination of the two.

There are several inherent limitations to our study. As are many other prostate cancer studies comparing treatment modalities, this study is not randomized. This potentially subjects the treatment groups to bias, although we attempted to adjust for these confounders in our multivariate analysis. Secondly, while we are able to compare patients treated with RP, EBRT, and BT, we did not have an adequate control group of patients with prostate cancer that did not go through treatment as this was not a common recommendation at the time that the study was started. We had only 10 patients that initially underwent observation in our cohort of patients, and many eventually opted for definitive treatment after a number of years. We also do not have data on ADT use or salvage treatments following the primary treatment, which would likely impact, at the very least, sexual function. As the use of ADT is usually used primarily with EBRT rather than RP or BT for initial treatment, this could unduly bias the results of our questionnaire, although most patients will not have received more than 3 years of ADT for definitive treatment, and the side effects of ADT should have dissipated by the time of this questionnaire over 10 years later. Finally, most of the participants were originally from a single geographical location, and were white, which reduces the generalizability of our study. Particularly, as African Americans have a higher incidence of prostate cancer, we would have liked to see if there were any race‐dependent differences in HRQOL.

Although our long follow‐up is highly valuable, over the years there have been many changes in techniques and technology. For example, many surgeons now perform nerve‐sparing radical prostatectomies, radiation oncologists use intensity‐modulated radiation therapy (IMRT), and there are improved planning techniques and postimplant evaluation for brachytherapy. Although it is questionable whether these advances have resulted in improved long‐term HRQOL, this is a confounding issue.

In summary, we report long‐term quality of life metrics in the urinary, bowel, and sexual function domains for patients following treatment for prostate cancer. This study will hopefully inform patients as to what to expect in their second decade of life after treatment, and help each person choose the best treatment option according to their personal needs and objectives.

## Conflict of Interest

The authors of this manuscript have no actual or potential conflicts of interest, except for: Jason Efstathiou: Advisory Board Member: Medivation/Astellas, Genentech; Joint Safety Review Committee: Bayer Healthcare. James Talcott: Consultant: Novartis.
